# Association of baseline negative life events and perceived social support with self-stigma in adolescents from Dongying, China

**DOI:** 10.3389/fpsyt.2026.1860158

**Published:** 2026-07-17

**Authors:** Rongrong Guo, Yuqi Yang, Yuelin Sun, Ruochen Ren, Xiaochuan Lu, Shengnan Liu, Chenxi Liu, Xi Wang, Xuan Zhao, Xiangdong Dong, Xiuli Ren, Yi Hu, Yufang Xing

**Affiliations:** 1 Department of Epidemiology and Health Statistics, School of Public Health, Qingdao University, Qingdao, China; 2 School of Nursing, Henan University of Science and Technology, Luoyang, Henan, China; 3 Yantai Center for Disease Control and Prevention, School Health Department, Yantai, China; 4 School of Public Health, Jilin University, Changchun, Jilin, China; 5 School of Medicine and Health Management, Tongji Medical School, Huazhong University of Science and Technology, Wuhan, China; 6 Dongying People’s Hospital, Dongying, China; 7 Dongying Shiyan Middle School, Dongying, China; 8 Dongying Dongcheng Hospital, Dongying, China; 9 School of Nursing, Shandong Second Medical University, Weifang, Shandong, China; 10 Shandong Center for Disease Control and Prevention, Business Management Department (Emergency Management Office), Jinan, China

**Keywords:** adolescents, gender, negative life events, perceived social support, self-stigma

## Abstract

**Background:**

Self-stigma can lead to many mental health issues. Negative life events (NLE) and perceived social support (PSS) are considered to be factors influencing self-stigma. This study aims to investigate gender-specific associations of NLE and PSS with self-stigma among Chinese adolescents.

**Methods:**

Data were obtained from an adolescent mental health surveillance conducted from September 2023 to September 2024. Self-stigma, NLE and PSS were measured repeatedly during three visits, with an average interval of six months. NLE and PSS were the main exposures. Generalized estimating equations were fitted to explore the individual and combined associations of NLE and PSS on self-stigma scores by gender.

**Results:**

A total of 4, 711 participants were included in the study, of whom 2, 252 were females. The study found that baseline NLE were associated with higher self-stigma scores in males (*β* = 0.036, *P* < 0.001), this was not significant in females (*β* = 0.019, *P* = 0.059). In addition, only found in females, low perceived social support (PSSL) and time at Visit3 (*χ*^2interaction^ = 9.05, *P* = 0.003), moderate perceived social support (PSSM) and time at Visit3 (*χ*^2interaction^ = 5.66, *P* = 0.017) had significant interactions with self-stigma. A comprehensive analysis indicated that participants who experienced NLE and PSSL had higher self-stigma scores (*β* = 0.199, *P* < 0.001 for males; *β* = 0.186, *P* < 0.001 for females).

**Conclusions:**

This study provides evidence that NLE and PSS have different associations on self-stigma in different genders.

## 1 Introduction

Self-stigma is defined as the process by which individuals internalize societal prejudice, absorbing public negative perceptions that eventually lead them to supplant their original social identity with a “disease identity” (1, 2). About four-fifths of people report experiencing stigma due to mental health issues, which is particularly prevalent among young people (3). Self-stigma leads to many poor mental health outcomes, including low self-esteem, self-absorption, lack of communication (4, 5). Adolescents are more vulnerable to mental health issues caused by discrimination, stigmatization, etc., which may lead to long-term adverse effects (6). Adolescence is a critical time for identity construction, during which self-stigma can limit students’ selves and lead to learned helplessness (7–9). In China, self-stigma among adolescents is common across multiple mental health issues, including depression-related family stigma (10), weight self-stigma linked to social anxiety (11, 12), and self-stigma among suicide attempters, lesbian, gay, and bisexual youth, and college students that mediates bullying and psychological distress (13–15).Understanding the important factors that contribute to self-stigma is crucial for preventing the deterioration of mental health problems, as well as developing early interventions for adolescents.

Previous studies on the impact of negative life events (NLE) on self-stigma were limited. During adolescence, some adolescents are confronted with NLE such as academic stress, relationships, and family changes (16). Some studies have reported the association between stress and stigmatization under different causes (17–19). Studies on the relationship between gender and self-stigma have reached conflicting conclusions. Stangl et al. reported that gender was significantly associated with stigmatization in studies adopting health stigma and discrimination frameworks (20). But Fond et al. reported that gender was not associated with self-stigma (21). However, few studies focus on the gender differences in the impact of NLE on self-stigma. Only a cross-sectional study conducted by Suen et al. found that after an increase in stressful life events, stigmatization increases significantly in males (22). There is a lack of longitudinal studies to confirm whether the impact of NLE on self-stigma differs by gender over time.

Social support is categorized into receiving social support and perceived social support (PSS). PSS refers to individuals’ belief in their ability to utilize social resources through social relationships (23, 24). Cross-sectional studies have found that high perceived social support (PSSH) is associated with lower stigma, while low perceived social support (PSSL) reinforces stigma (25, 26). However, to date, few studies have investigated whether this relationship remains consistent across genders over time. Furthermore, the combined impact of NLE and PSS on self-stigma also requires further investigation.

This study utilized data from a longitudinal survey and employed Generalized Estimating Equations (GEE) to explore the gender-specific associations of NLE and PSS with long-term changes in self-stigma, as well as determine the combined effects of NLE and PSS on self-stigma in adolescents.

## 2 Methods

### 2.1 Participants and procedures

Data were obtained from the Adolescent Mental Health Stigma Monitoring Cohort Study. The Adolescent Mental Health Stigma Monitoring Cohort Study targets middle school students from Dongying Experimental Middle School. Its primary objective is to evaluate adolescent mental health and identify its influencing factors. All classes were invited to participate using classroom-based cluster sampling. Data collection platform and timeline. Study information was collected through an electronic questionnaire administered via the Wenjuanxing platform. The baseline survey began in September 2023. Data from September 2023 (Visit 1), March 2024 (Visit 2), and September 2024 (Visit 3) were included in this study. Each wave of data collection was completed within one month. No monetary or course credit incentives were provided to participants. Ethics and informed consent. The study was approved by the Ethics Committee for Biomedical Research Involving Human Beings. The participants themselves and their guardians co-signed an informed consent form.

Inclusion criteria before data collection. Participants had to: (a) be a registered student in Grades 6 to 8 at Dongying Experimental Middle School (the study spanned one academic year from September 2023 to September 2024; Grade 9 students were excluded because they would graduate in June 2024, making follow−up difficult); (b) be able to understand and complete the electronic questionnaire independently; and (c) provide signed informed consent from both themselves and their legal guardians. Before data collection, students who were on long−term leave, suspended from school, or had diagnosed cognitive impairments that prevented independent completion of the questionnaire were not invited. Recruitment and data collection. All eligible students who met the inclusion criteria and were invited received an informed consent form. Students whose guardians did not sign the consent were not allowed to participate. All invited students who provided signed consent completed the electronic questionnaire. Data were cleaned to exclude ineligible questionnaires using the following objective criteria: 1. Missing or incorrect ID: The student ID number did not follow the required 8−digit format (8−digit number: year + class + personal number, e.g., 20230101), contained non−numeric characters, was left blank, or was a duplicate of another participant’s ID. 2. Careless responding (“filled out in the same way”): The participant selected the same response option (e.g., all “agree” or all “3−neutral”) for all items of any scale or for the entire questionnaire. 3. Logical errors: Reported age ≥ 20 years or other clearly impossible values. 4. Duplicate submissions: The same participant submitted more than one questionnaire.

After applying these criteria, 4, 711 valid questionnaires remained. These 4, 711 participants constituted the baseline analytic sample. Among the 4, 711 baseline participants, 2, 252 were females and 2, 459 were males. Follow−up and retention for Visit 2 and Visit 3. Of these 4, 711 participants, 3, 417 (72.5%) completed Visit 2 and 3, 155 (67.0%) completed Visit 3. To retain participants, research staff conducted telephone follow−ups with non−respondents and coordinated with homeroom teachers to remind students. Participants who missed a visit were not replaced; all available data were used under the missing at random assumption in the generalized estimating equations. No incentives were provided for completion of follow−up visits.

### 2.2 Measures

#### 2.2.1 Internalized stigma of mental illness scale (ISMI-10)

Self-stigma was measured using the Internalized Stigma of Mental Illness scale (ISMI-10). The ISMI-10 consists of 10 items rated on a 5-point Likert scale from 1 (strongly disagree) to 5 (strongly agree), with items 2 and 9 reverse-coded. Total scores range from 10 to 50, with higher scores indicating higher levels of self-stigma. Previous research on this scale has had high construct validity and internal consistency (27). In this study, the Cronbach’s alpha coefficients of the scale were 0.815, 0.839, and 0.853 at Visit 1, Visit 2, and Visit 3.

#### 2.2.2 Negative life events

NLE were collected through self-reported questionnaires at baseline. The questionnaire included six types of NLE: 1. Frustration in interpersonal relationships (including discrimination, misunderstanding, public humiliation, and conflicts with classmates or friends); 2. Academic stress (including exam failure, heavy study burden, failure in honors evaluation, and academic promotion pressure); 3. Family disruptions (including major family illness/death, intrafamily conflicts, financial hardship, and prolonged separation); 4. Punishment (including parental physical/verbal abuse, school criticism/discipline, and violation of discipline/law); 5. Serious personal illness and accidental injuries; 6. Romantic relationship difficulties or breakup. If participants did not experience any NLE, they were scored as 0 (NLE0); if participants experienced at least one NLE, they were scored as 1 (NLE1).

#### 2.2.3 Multidimensional scale of perceived social support

PSS was measured using the multidimensional scale of perceived social support (MPSS). This was a 12-item scale measured on a 5-point Likert scale. Total scores range from 12 to 60, with higher scores indicating greater perceived social support. Total scores range from 12 to 60, with higher scores indicated more PSS. The validity and reliability of this scale have been carefully evaluated (28). Based on PSS tertiles, participants were divided into low perceived social support (PSSL), medium perceived social support (PSSM), and high perceived social support (PSSH). Cronbach’s alpha coefficients for this scale were 0.964, 0.971, and 0.977 at Visit1, 2, and 3.

#### 2.2.4 Covariates

Covariates were obtained from the questionnaire at Visit 1. Demographic characteristics included in the baseline questionnaire were age (years), only-child status (no=0, yes=1), family size, primary caregiver (mother=1, father=2, both parents together=3, grandparents=4, other=5), and household income (less than 100, 000 yuan=1; 100, 000 yuan-200, 000 yuan=2; 200, 000 yuan-300, 000 yuan=3; 300, 000 yuan or more=4).

### 2.3 Statistical analysis

Quantitative variables were reported as median (interquartile range). Qualitative variables were reported as frequency (%). Quantitative variables were analyzed using the Mann-Whitney U test, qualitative variables were analyzed using the chi-square test, and ordinal variables were analyzed using the Kruskal-Wallis H test.

Generalized estimating equations (GEE) were employed to analyze longitudinal associations of NLE and PSS with self-stigma. Because not all participants completed all three visits, GEE can accommodate unbalanced repeated measures under the missing at random (MAR) assumption, using all available data from all 4, 711 participants. Age, only-child status, family size, primary caregiver, and household income were included in the analytical model. The model was constructed based on the skewed distribution characteristics of the data. Multiple models were built to compare the Quasi-Likelihood Information Criterion (QIC) values for different combinations of distributions and link functions. Finally, the gamma distribution, log-link function, and exchangeable correlation structure were selected to construct the model, which fits the data characteristics. The *geeglm* function of the *geepack* package was used to fit the model. The Wald test (*α* = 0.05) was performed to assess parameter significance for the interaction effect. All analyses were done in R 4.2.1.

## 3 Results

### 3.1 Descriptive analysis

Demographic information was presented in **Table 1**. A total of 2, 459 males and 2, 252 females were enrolled at baseline, with a mean age of 12 years (IQR = 1). At baseline, 41.6% of participants reported experiencing NLE, while 58.4% reported not experienced NLE. At baseline, 42.9% of males and 46.2% of females reported experiencing NLE. For PSS, at baseline about 32.3% males and 35.8% females reported PSSL. See **Table 1** for detailed information on NLE and PSS for Visit2 and Visit3.

**Table 1:** Descriptive statistics of the study sample at each visit, stratified by gender.

Characteristics	Male	Female
Age, mean ± IQR	12 ± 1	12 ± 1
Only-child status, %		
No	70.4	79.8
Yes	29.6	20.2
Family Size, mean ± IQR	4 ± 1	4 ± 0
Primary Caregiver, %		
Mother	22.4	21.8
Father	2.4	1.7
Both parents together	73.1	74.0
Grandparents	1.5	1.7
Other	0.6	0.8
Household income, %		
Less than 100, 000 yuan	24.6	24.9
100, 000 yuan-200, 000 yuan	38.7	38.5
200, 000 yuan-300, 000 yuan	22.6	22.6
300, 000 yuan or more	14.1	14.1
Perceived social support, %		
High (Visit 1)	31.2	30.7
Medium (Visit 1)	36.5	33.5
Low (Visit 1)	32.3	35.8
High (Visit 2) ^#^	32.3	30.6
Medium (Visit 2) ^#^	34.0	31.9
Low (Visit 2) ^#^	33.7	37.6
High (Visit 3) ^#^	30.4	32.1
Medium (Visit 3) ^#^	32.1	29.0
Low (Visit 3) ^#^	37.6	38.9
Negative life events, %		
No (Visit 1)	57.1	53.8
Yes (Visit 1)	42.9	46.2
No (Visit 2) ^#^	59.5	53.3
Yes (Visit 2) ^#^	40.5	46.7
No (Visit 3) ^#^	59.2	54.0
Yes (Visit 3) ^#^	40.8	46.0
Self-stigma, mean ± IQR		
Visit 1	26 ± 8	26 ± 8
Visit 2^#^	27 ± 8	26 ± 8
Visit 3^#^	27 ± 8	27 ± 8

1. Age, family size, and self-stigma are presented as mean ± IQR.; 2. Only-child status, primary caregiver, household income, perceived social support, and negative life events are presented as frequency (%).; 3. Demographic characteristics (age, only-child status, family size, primary caregiver, household income) were assessed at baseline (Visit 1) and do not vary over time. IQR, interquartile range; %, percentage. ^#^ Percentages for Visit 2 and Visit 3 are based on the number of participants who completed each respective visit (Visit 2: N = 3, 417; Visit 3: N = 3, 155).

### 3.2 Association between baseline NLE and self-stigma over time

**Figure 1:**
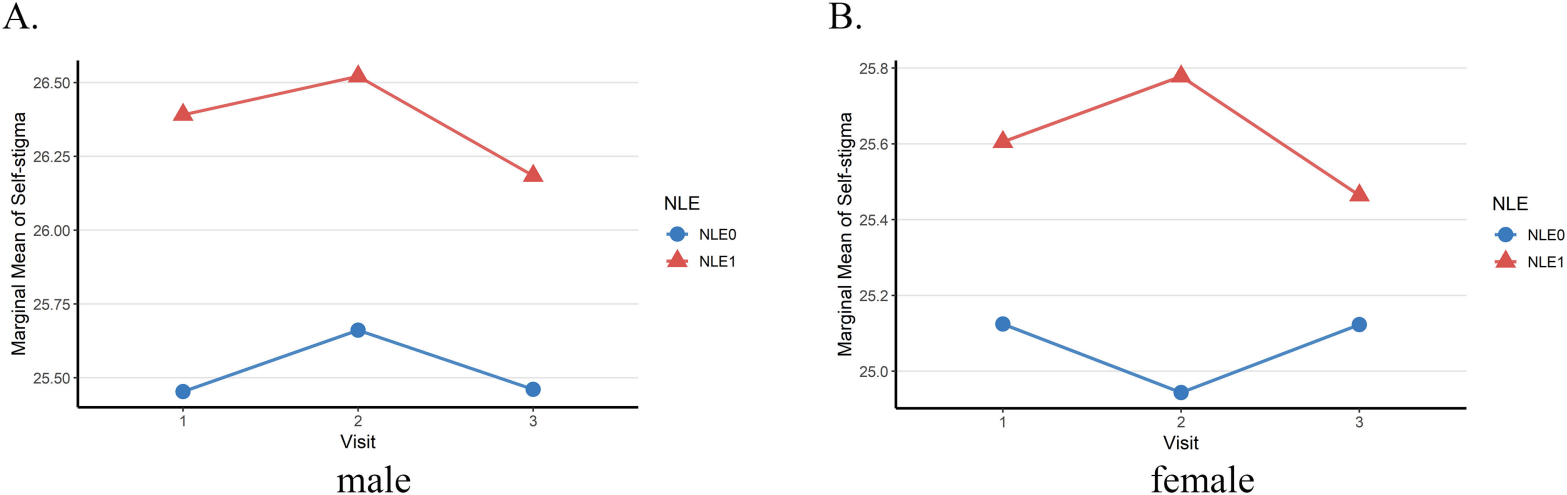
The independent effects of negative life events and perceived social support on self-stigma scores over time in males and females. X-axis: Study visit (Visit 1, Visit 2, Visit 3), representing the three time points of measurement; Y-axis: Marginal mean of self−stigma scores, representing the level of self−stigma for each group over time; NLE, negative life events; PSS, perceived social support; NLE0, not experiencing a negative life event; NLE1, experiencing at least one negative life event; PSSH, high perceived social support; PSSM, moderate perceived social support; PSSL, low perceived social support. **(A)** Male participants; **(B)** Female participants.

The results of the GEE analysis of NLE and time on self-stigma were shown in **Table 2**. GEE was fitted to assess the association between NLE and self-stigma. In male participants, those who had experienced NLE scored significantly higher on self-stigma than those who had not experienced NLE (*β* = 0.036, *P* < 0.001). However, in female participants, there was no significant difference in self-stigma scores between those who had experienced NLE and those who had not experienced NLE (*β* = 0.019, *P* = 0.059). No significant interaction between NLE and time on self-stigma was found in male (joint test: *χ*^2interaction^ =0.304, df = 2, *P* = 0.859) or female participants (joint test: *χ*^2interaction^ =1.757, df = 2, *P* = 0.415), indicating that the association of NLE on self-stigma did not increase or decrease over time. As shown in **Figure 1**, in both male and female participants, self-stigma scores were higher in the group that experienced NLE than in the group that did not experience NLE at the Visit 1, 2, and 3.

**Table 2:** Generalized estimating equations were used to analyze the association between negative life events and perceived social support and self-stigma.

		Male		Female	
		*β* (95% CI)	*P*-value	*β* (95% CI)	*P*-value
NLE		0.036 (0.017 to 0.055)	< 0.001^***^	0.019 (-0.001 to 0.039)	0.059
Perceived social support	Middle	0.106 (0.080 to 0.131)	< 0.001^***^	0.082 (0.055 to 0.108)	< 0.001^***^
	Low	0.160 (0.136 to 0.185)	< 0.001^***^	0.156 (0.130 to 0.182)	< 0.001^***^
Time	Visit2	0.022 (-0.013 to 0.056)	0.217	-0.019 (-0.050 to 0.012)	0.237
	Visit3	0.002 (-0.032 to 0.036)	0.921	-0.036 (-0.070 to -0.002)	**0.037** ^*^
NLE × Time	NLE × Visit2	-0.003 (-0.034 to 0.027)	0.836	0.014 (-0.014 to 0.042)	0.333
	NLE × Visit3	-0.008 (-0.037 to 0.021)	0.584	-0.005 (-0.036 to 0.025)	0.721
Perceived social support × Time	PSSM × Visit2	-0.030 (-0.070 to0.009)	0.132	0.030 (-0.008 to 0.067)	0.113
	PSSM × Visit3	-0.021 (-0.060 to 0.018)	0.298	0.048 (0.009 to 0.089)	**0.017** ^*^
	PSSL × Visit2	-0.010 (-0.047 to 0.027)	0.597	0.004 (-0.032 to 0.041)	0.820
	PSSL × Visit3	0.017 (-0.020 to 0.054)	0.380	0.059 (0.021 to 0.098)	0.003^**^

*β*, beta coefficient; 95% CI, 95% confidence interval; NLE, negative life event; PSSM, medium perceived social support; PSSL, low perceived social support. Exposure × Time: Interaction term between exposure duration and time.

^*^ *P* < 0.05, ^**^P < 0.01, ^***^P < 0.001.

Bolded P-values indicate statistically significant associations.

### 3.3 Association between PSS and self-stigma over time

**Figure 2:**
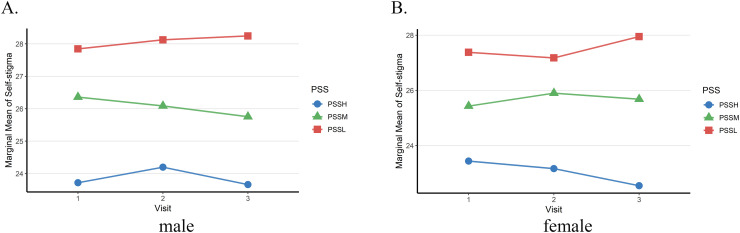
Combined effects of negative life events and perceived social support with self-stigma scores over time. X-axis: Study visit (Visit 1, Visit 2, Visit 3), representing the three time points of measurement; Y-axis: Marginal mean of self−stigma scores, representing the level of self−stigma for each group over time; NLE0, not experiencing a negative life event; NLE1, experiencing at least one negative life event; PSSH, high perceived social support; PSSM, moderate perceived social support; PSSL, low perceived social support. **(A)** Male participants; **(B)** Female participants.

The results of the GEE analysis of PSS and time on self-stigma were shown in **Table 2**. Compared with participants with PSSH, male and female participants with PSSL (*β* = 0.160, *P* < 0.001 for males and *β* = 0.156, *P* < 0.001 for females) and PSSM (*β* = 0.106, *P* < 0.001 for males and *β* = 0.082, *P* < 0.001 for females) had higher self-stigma scores. In male participants, no significant interaction between PSS and time was found in terms of self-stigma (joint test: *χ*^2interaction^ = 8.204, df = 4, *P* = 0.084). However, among female participants, we found that compared with the PSSH group, the PSSL group (*χ*^2interaction^ = 9.05, *P* = 0.003) and PSSM group (*χ*^2interaction^ = 5.66, *P* = 0.017) had an interaction with time at Visit 3 on self-stigma. This indicated that the association of PSSM and PSSL on self-stigma among females is time-dependent and more pronounced with long-term exposure. Participants with PSSH had the lowest self-stigma scores at Visit 1, Visit 2, and Visit 3 (**Figure 2**).

### 3.4 Combined associations of NLE and PSS with self-stigma over time

**Figure 3:**
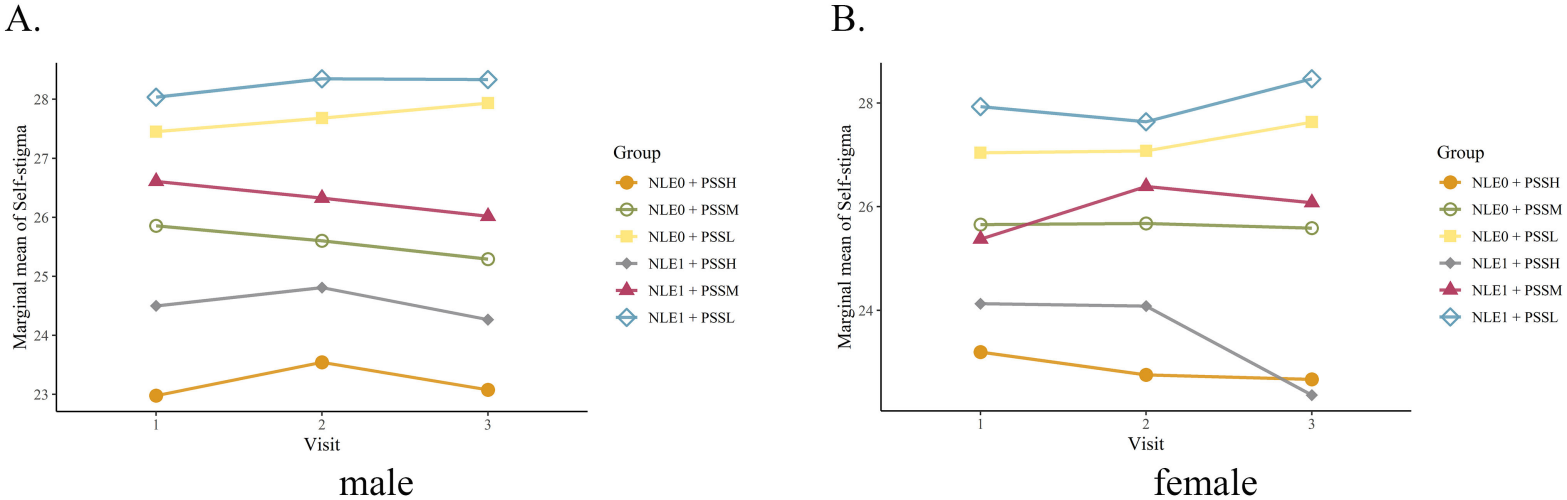
Combined effects of negative life events and perceived social support on self-stigma scores across visits, stratified by gender. X-axis: Study visit (Visit 1, Visit 2, Visit 3), representing the three time points of measurement; Y-axis: Marginal mean of self−stigma scores, representing the level of self−stigma for each group over time; NLE0, not experiencing a negative life event; NLE1, experiencing at least one negative life event; PSSH, high perceived social support; PSSM, moderate perceived social support; PSSL, low perceived social support. **(A)** Male participants; **(B)** Female participants.

The combined associations of NLE and PSS with self-stigma were shown in **Table 3**. Among male participants, combined exposure was associated with higher self-stigma scores. However, among female participants, there was no significant difference in self-stigma scores (*β* = 0.039, *P* = 0.108) between the NLE1 + PSSH group and NLE0 + PSSH group. Female participants with PSSH, experienced NLE and did not experience NLE had no effect on self-stigma scores. As shown in **Figure 3**, regardless of whether the participants were male or female, the self-stigma scores of the NLE1 + PSSL group were the highest in all three visits.

**Table 3:** Generalized estimating equations were used to analyze the joint effects of negative life events and perceived social support on self-stigma over time.

	Male		Female	
	*β* (95% CI)	*P*-value	*β* (95% CI)	*P*-value
Combinations				
NLE0 + PSSM	0.118 (0.087 to 0.149)	< 0.001^***^	0.101 (0.069 to 0.133)	< 0.001^***^
NLE0 + PSSL	0.178 (0.147 to 0.209)	< 0.001^***^	0.153 (0.122 to 0.185)	< 0.001^***^
NLE1 + PSSH	0.064 (0.019 to 0.109)	0.005^**^	0.039 (-0.009 to 0.087)	0.108
NLE1 + PSSM	0.147 (0.112 to 0.181)	< 0.001^***^	0.090 (0.055 to 0.124)	< 0.001^***^
NLE1 + PSSL	0.199 (0.168 to 0.230)	< 0.001^***^	0.186 (0.154 to 0.217)	< 0.001^***^
Combinations × time				
High perceived social support				
(NLE1 + PSSH) × Visit 2	-0.012 (-0.084 to 0.061)	0.753	0.017 (-0.051 to 0.085)	0.619
(NLE1 + PSSH) × Visit 3	-0.014 (-0.085 to 0.057)	0.701	-0.053 (-0.125 to 0.019)	0.152
Medium perceived social support				
(NLE0 + PSSM) × Visit 2	-0.034 (-0.082 to 0.014)	0.163	0.020 (-0.024 to 0.065)	0.375
(NLE0 + PSSM) × Visit 3	-0.026 (-0.074 to 0.021)	0.279	0.020 (-0.028 to 0.069)	0.412
(NLE1 + PSSM) × Visit 2	-0.035 (-0.089 to 0.019)	0.207	0.058 (0.010 to 0.107)	**0.017** ^*^
(NLE1 + PSSM) × Visit 3	-0.027 (-0.081 to 0.028)	0.335	0.050 (-0.003 to 0.103)	0.062
Low perceived social support				
(NLE0 + PSSL) × Visit 2	-0.016 (-0.061 to 0.030)	0.493	0.021 (-0.024 to 0.065)	0.365
(NLE0 + PSSL) × Visit 3	0.013 (-0.033 to 0.059)	0.578	0.045 (-0.003 to 0.092)	0.066
(NLE1 + PSSL) × Visit 2	-0.013 (-0.060 to 0.033)	0.574	0.009 (-0.035 to 0.052)	0.691
(NLE1 + PSSL) × Visit 3	0.006 (-0.039 to 0.052)	0.789	0.042 (-0.004 to 0.089)	0.076

NLE0, not experiencing a negative life event; NLE1, experiencing at least one negative life event; PSSH, high perceived social support; PSSM, moderate perceived social support; PSSL, low perceived social support; *β*, beta coefficient; 95% CI, 95% confidence interval. Exposure × Time: Interaction term between exposure duration and time.

^*^ *P* < 0.05, ^**^P < 0.01, ^***^P < 0.001.

Bolded P-values represent results with statistical significance.

## 4 Discussion

This prospective study aimed to assess the independent and combined associations of baseline NLE and repeated measures of PSS with self-stigma among Chinese adolescents. The main findings were as follows: 1) The results showed that baseline NLE were associated with higher self-stigma scores in males, while NLE had no significant association with self-stigma in females; 2) Compared with the PSSH group, the PSSL and PSSM groups were associated with higher self-stigma scores in both genders; 3) The interaction between PSS and time was only found in female participants, with PSSL and PSSM showing stronger associations with self-stigma at Visit 3; 4) Most combinations of NLE and PSS were associated with higher self-stigma scores in both genders, except for females with NLE and high PSS.

NLE refers to stressful events faced by adolescents in interpersonal relationships, academics, family, school, etc. (29, 30). The present study found a gender differences in the association between NLE and self-stigma. NLE was associated with self-stigma, but this association was not significant in females. Our research was consistent with the findings of many previous studies conducted in adults. A cross-sectional study found a significant correlation between stigma and stress in both male and female populations (17). Suen et al. found that in men an increase in stress events increases the risk of stigmatization, but in women no such association was found (22). Results from an observational multicenter study involving 17 European countries also showed that compared with females, perceived stigma is more likely to be associated with males (31). From the perspective of Chinese cultural context, collectivist values and face concern may reinforce this process: in highly collectivist cultures, the link between external stress and self-stigma is stronger (32). Face concern not only directly exacerbates self-stigma but also mediates the relationship between public stigma and self-stigma (33, 34). Moreover, traditional gender role expectations place higher family and academic pressures on males, which may explain why negative life events have a stronger impact on self-stigma in males (35). Previous studies were consistent with our findings. However, there have been no longitudinal studies exploring the association between NLE and self-stigma in adolescents so far.

The relationship between social support and stigma has been extensively studied (36, 37). For example, a study of adult college students found a significant relationship between low social support and self-stigma (36). Similarly, another study found that participants with better social support were less to be stigmatized (37). These findings were consistent with our research findings, which showed that PSSM and PSSL were associated with higher self-stigma scores compared to PSSH. However, our study also found that females who experienced PSSM and PSSL for a longer period of time had higher self-stigma scores. This was consistent with a longitudinal study finding that social support predicts stigmatization one year later (38). But there was a lack of longitudinal studies to verify whether the interaction between PSS and time has different effects on self-stigma in different genders.

The exact mechanisms by which NLE and PSS influence self-stigma were unclear. However, seeking help may be a potential psychosocial mechanism for self-stigma in NLE and PSS. A previous study found that stress from life events is associated with seeking help (39). Similarly, a prospective survey among adolescents found that stressful life events were associated with help-seeking (40). Zhang et al., found that lower perceived social support was associated with an attitude of unwillingness to seek help (41). A literature review study also showed that for children and adolescents, academic problems, life events, and other factors are the determinants of their help-seeking (42). In the results of a systematic review, it was found that internalized stigma is associated with a decrease in help-seeking willingness (43). In European countries, it was also found that in regions with high help-seeking rates, the level of stigmatization was lower (44).

Our study also found that among female participants with PSSH, there was no significant difference in self-stigma scores between those who experienced NLE and those who did not. In contrast, all other combinations across participants led to increased self-stigma scores. In both male and female participants, the remaining groups showed significantly higher self-stigma scores than the control group. Previous studies on the combined effects of NLE and PSS on self-stigma have conflicting conclusions. A study of community populations indicated that perceived social support was associated with stress, but perceived social support did not mitigate the adverse effects of stress on disease (45). In studies on the extent of depression, it was found that only adverse life events played a role in the partial remission group of depression, whereas in the major depression group, social support was an important factor (46). In contrast, high perceived social support was not predictive post-traumatic stress disorder (47). The results of a ten-year follow-up study suggested that social support only works when exposed to stress (48). Therefore, we inferred that NLE and PSS may have played different roles in various mental health problems, and that gender differences might have existed.

Strengths of this study: 1) Longitudinal study data from Chinese adolescents were used. 2) In addition to individual effects, it explored combined effects across different genders using GEE. However, several limitations should be acknowledged. First, although generalized estimating equations (GEE) used all available data under the missing at random assumption, there were still participants lost to follow-up, and a complete-case analysis alone may introduce bias. Second, negative life events were measured only at baseline; therefore, we could not examine how changes in life events over the follow-up period might influence self-stigma, nor can we infer causality. Third, the sample was drawn from a single school, which limits the generalizability of the findings. Fourth, although the ISMI-10 scale showed good internal consistency in our sample, it has not been extensively validated in general adolescent populations in China. Future research should include multi-site samples, measure life events repeatedly, and employ causal inference methods.

## 5 Conclusions

In summary, there are differences in the association between NLE and self-stigma across genders, with higher self-stigma scores observed in both males and females who experienced NLE and PSSL. The study supports the development of gender-specific intervention measures for self-stigma among adolescents.

## Data Availability

The raw data supporting the conclusions of this article will be made available by the authors, without undue reservation.
